# Inhibition of FGF/FGFR autocrine signaling in mesothelioma with the FGF ligand trap, FP-1039/GSK3052230

**DOI:** 10.18632/oncotarget.9515

**Published:** 2016-05-20

**Authors:** Christina Blackwell, Christian Sherk, Maggie Fricko, Gopinath Ganji, Mary Barnette, Bao Hoang, James Tunstead, Tina Skedzielewski, Hasan Alsaid, Beat M. Jucker, Elisabeth Minthorn, Rakesh Kumar, M. Phillip DeYoung

**Affiliations:** ^1^ Oncology R&D, GlaxoSmithKline Research and Development, Collegeville, PA 19426, USA; ^2^ Platform Technology and Science, GlaxoSmithKline Research and Development, King of Prussia, PA 19406, USA

**Keywords:** fibroblast growth factor, ligand trap, mesothelioma, signaling, angiogenesis

## Abstract

Fibroblast growth factor (FGF) ligand-dependent signaling has a fundamental role in cancer development and tumor maintenance. GSK3052230 (also known as FP-1039) is a soluble decoy receptor that sequesters FGFs and inhibits FGFR signaling. Herein, the efficacy of this molecule was tested in models of mesothelioma, a tumor type shown to express high levels of FGF2 and FGFR1. GSK3052230 demonstrated antiproliferative activity across a panel of mesothelioma cell lines and inhibited growth of tumor xenografts in mice. High expression of FGF2 and FGFR1 correlated well with response to FGF pathway inhibition. GSK3052230 inhibited MAPK signaling as evidenced by decreased phospho-ERK and phospho-S6 levels *in vitro* and *in vivo*. Additionally, dose-dependent and statistically-significant reductions in tumor vessel density were observed in GSK3052230-treated tumors compared to vehicle-treated tumors. These data support the role of GSK3052230 in effectively targeting FGF-FGFR autocrine signaling in mesothelioma, demonstrate its impact on tumor growth and angiogenesis, and provide a rationale for the current clinical evaluation of this molecule in mesothelioma patients.

## INTRODUCTION

The fibroblast growth factor (FGF) signaling pathway is involved in the initiation, establishment and growth of tumors across multiple histologies [1, 2]. The pathway plays many roles in the development of cancer, including regulation of cell growth and differentiation, regulation of angiogenesis, and participation in tumor-stroma interactions. The FGF proteins are a large family of 18 distinct secreted growth factors that bind to and activate a family of 4 FGF receptors (FGFRs) and 4 structurally related non-receptor proteins [3]. A number of FGF/FGFR genomic alterations have been identified and are largely cancer specific, but the mechanisms by which they drive FGF signaling can be classified as ligand-independent (receptor gene amplifications, mutations, gene fusions) or ligand-dependent (ligand and/or receptor gene amplifications, overexpression, alternative splicing, autocrine and paracrine signaling) [2]. With regards to ligand-dependent signaling, it has been shown that FGFR1-amplified tumor cells require FGF ligands for signaling and growth, and that overexpression of FGFs or coexpression of both FGFs and FGFRs occur in non small cell lung cancer (NSCLC), head and neck squamous cell carcinomas (HNSCC), and in basal-like breast cancer cells [4–7]. FGF-FGFR signaling is also known to be implicated in drug resistance to targeted therapies directed at other receptor tyrosine kinases [8].

GSK3052230 is a soluble fusion protein consisting of the extracellular domains of the human FGFR1 α-IIIc isoform linked to the modified hinge and native Fc regions of human immunoglobulin G1 (IgG1). GSK3052230 acts as a fusion protein “trap” that sequesters FGFs, neutralizing their ability to bind to and activate FGFRs, particularly FGFR1. The FGFR1 α-IIIc isoform was chosen to form the trap domain of GSK3052230 as it has the broadest ligand binding profile of the FGFR1 isoforms and does not bind the hormonal FGFs (FGF19, FGF21, and FGF23) with high affinity [9–11]. Thus, the unique FGF binding profile of GSK3052230 should avoid the potential on-target toxicities associated with small molecule pan FGFR kinase inhibitors, such as hyperphosphatemia and retinal, nail, and skin changes [reviewed in ref. 12]. GSK3052230 has been shown to inhibit tumor growth in several cell line-derived xenograft and patient-derived xenograft (PDX) tumor models, including FGFR1-amplified lung cancer and FGFR2-mutated endometrial cancer models, and response to GSK3052230 positively correlated with overexpression of FGF2, FGF18, FGFR1c, FGFR3c, and ETV4 RNA levels [13].

Malignant pleural mesothelioma (MPM), an asbestos-related cancer that develops in the membrane lining of the lungs and abdomen, remains a deadly disease with few effective therapies. Although the incidence of mesothelioma is leveling off in the United States, the incidence in Western Europe, China, Russia, and India continues to rise [14]. The standard of care for front-line treatment remains cisplatin and pemetrexed with the combination regimen having a 41% response rate, a median time to progression of 5.7 months, and a median overall survival of 12.1 months [15]. For recurrent disease, there remains no widely approved regimen although a number of chemotherapeutic agents that have been used with limited success [16–18]. These data underscore the need for more effective therapies in mesothelioma. Here, we provide evidence using a targeted therapy approach in several preclinical models that mesothelioma cell lines and tumors are particularly sensitive to inhibition of FGF-FGFR autocrine signaling by GSK3052230.

## RESULTS

### GSK3052230 inhibits growth of FGF2/FGFR1-overexpressing mesothelioma cells

**Figure 1:**
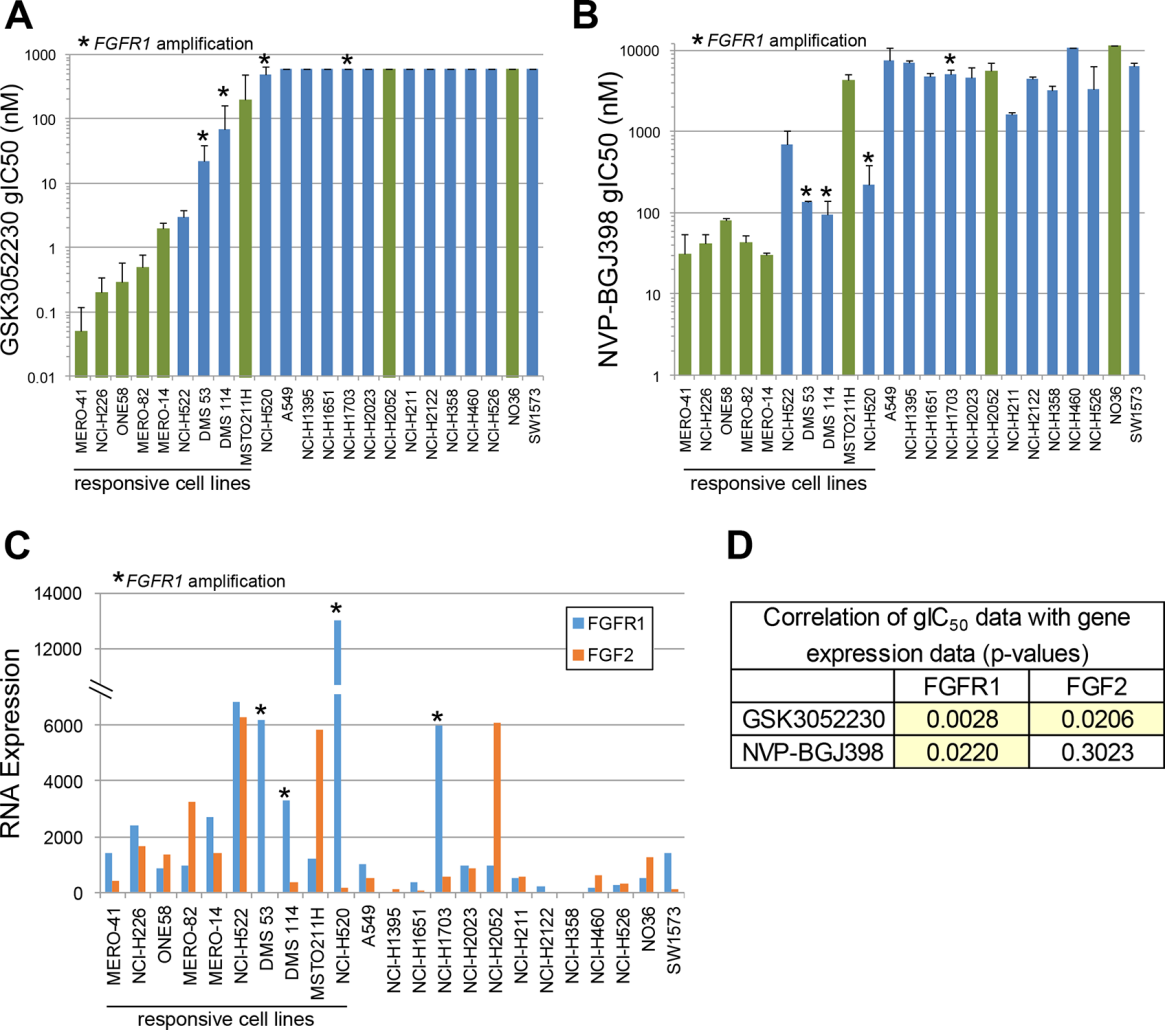
FGF2 and FGFR1 RNA overexpression correlates with response to FGF/FGFR inhibitors in mesothelioma and lung cancer cell lines Cells were treated with GSK3052230 (**A**) or NVP-BGJ398 (**B**) for 6 days in anchorage-independent methylcellulose (AIMC) media. Growth IC_50_ values (gIC_50_) represent the concentration that inhibited cellular growth by 50% (midpoint of the growth window between cells at plating and the growth of control cells at day 6). Bars represent the mean gIC_50_ values of duplicate samples from two independent experiments. Green bars: mesothelioma cells; blue bars: lung cancer cells. Error bars correspond to the standard deviation. * denotes cell lines that harbor FGFR1 amplification. (**C**) Cells grown in AIMC media for 6 days were harvested for RNA and relative expression levels for the FGF family were explored (FGF2 and FGFR1 RNA expression shown here). (**D**) Correlation of GSK3052230 and NVP-BGJ398 gIC_50_ data with FGFR1 and FGF2 gene expression data. *p*-values were calculated using Spearman's rank correlation.

Overexpression of FGF2 protein has been observed in primary mesothelioma tumor specimens [19–22], and both FGF2 and FGFR1 mRNA expression levels are high in mesothelioma compared to other tumor types in The Cancer Genome Atlas (TCGA) collection (Supplementary Figure 1). Similarly, FGF2 mRNA levels are highest in mesothelioma cell lines compared to all other cancer cell lines in the Cancer Cell Line Encyclopedia (CCLE) [23], and FGFR1 expression is also high in these cells (Supplementary Figure 2). We hypothesized that by trapping ligands of the FGF family, GSK3052230 will inhibit tumor cell proliferation and/or inhibit tumor angiogenesis. To test this, two FGF pathway inhibitors, GSK3052230 and NVP-BGJ398 [24], a small molecule pan FGFR kinase inhibitor, were screened in an anchorage-independent methylcellulose (AIMC) assay for their effects on the growth of a panel of 23 mesothelioma and lung cancer cell lines spanning various histologies. Antiproliferative activity was observed across the majority of mesothelioma cells (6/8) and in a few lung cancer cells lines (4/15) with both molecules (Figure 1A and 1B). Three of the sensitive lung cancer cell lines, DMS 53, DMS 114, and NCI-H520, harbored FGFR1 amplification and were previously shown to respond to GSK3052230 treatment [13]. The fourth cell line, NCI-H522, is a lung adenocarcinoma cell line that does not contain any FGFR genomic alterations. With the exception of NCI-H520 and MSTO-211H, there was very good overlap across the entire cell line panel with regards to antiproliferative response to both molecules.

Previous work demonstrated that FGFR1 genomic amplification and increased FGF2 mRNA levels correlated with response to GSK3052230 in tumor xenograft models [13]. To determine if expression of other FGF family members correlates with response to FGF pathway inhibition, baseline RNA levels of the entire FGF family (22 ligands and 4 receptors) were assayed in this cell line panel. FGF2 and FGFR1 were the most highly and broadly expressed FGF ligand and receptor, respectively, across the cell lines (Supplementary Figure 3). Relative to FGF2 and FGFR1, most other family members showed little or no (below the limit of detection) expression across the panel. When RNA expression data for these genes were compared with GSK3052230 or NVP-BGJ398 gIC_50_ data, high levels of FGF2 and FGFR1 correlated well with response to both compounds (Figure 1C). Statistically, FGFR1 expression correlated better with response to both FGF pathway inhibitors than FGF2 expression (Figure 1D). This data is consistent with prior work demonstrating that FGFR1 expression could be used as a biomarker in lung cancer and mesothelioma [25, 26]. Taken together, this data suggests that FGF autocrine signaling based on FGF2 and/or FGFR1 overexpression is important for mesothelioma cell proliferation.

### Effects of GSK3052230 on FGF pathway signaling *in vitro*

**Figure 2:**
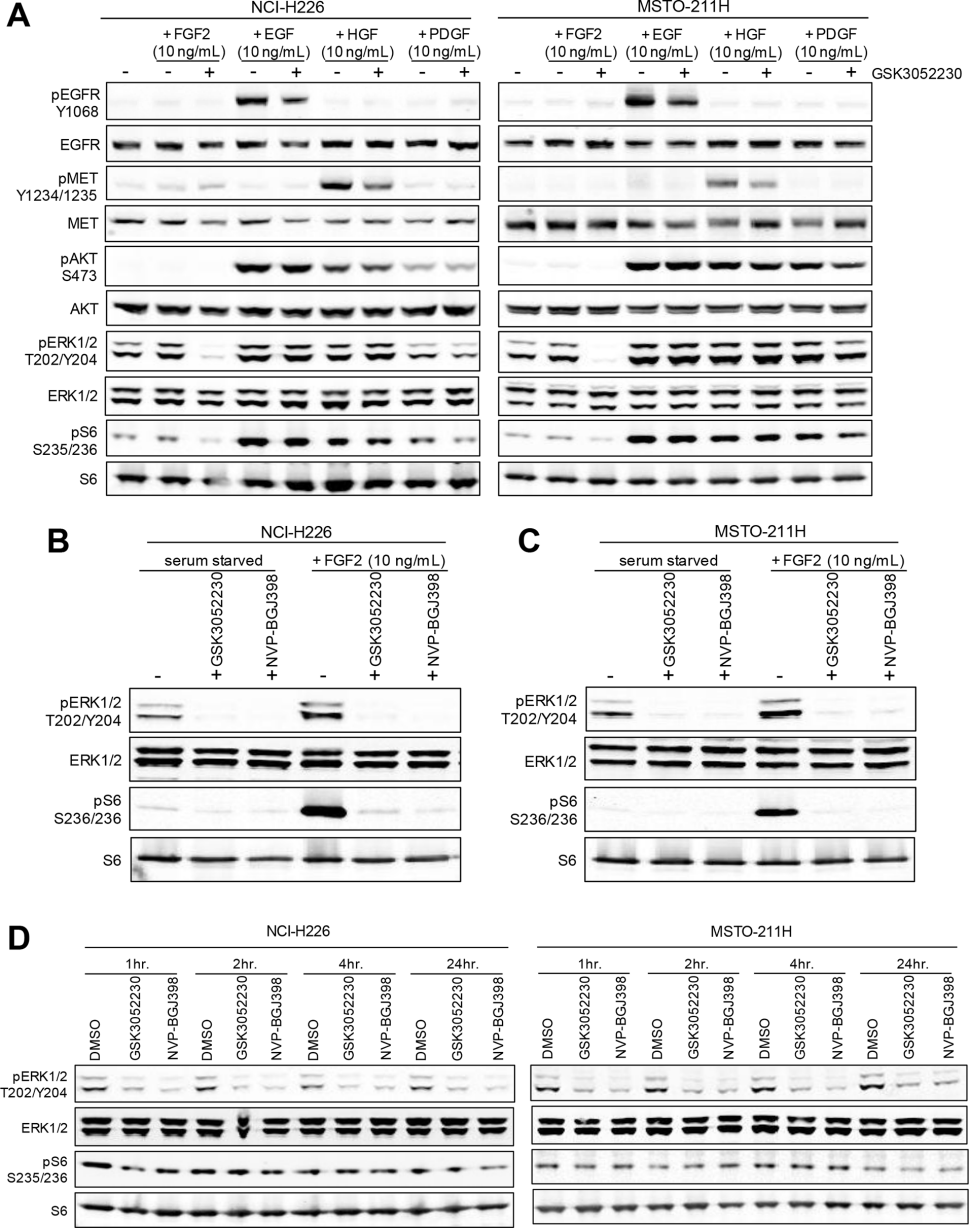
PI3K/AKT and MAPK signaling after growth factor stimulation in mesothelioma cell lines (**A**) NCI-H226 and MSTO-211H cells were serum starved for 24 hours and then pretreated with heparin sodium salt (10 μg/mL) with or without GSK3052230 (15 μg/mL) for 2 hours prior to the addition of growth factors for 15 minutes. Protein lysates were harvested and subjected to western blot analysis. (**B** and **C**) GSK3052230 (15 μg/mL) and NVP-BGJ398 (500 nM) treatment inhibited phospho-ERK and phospho-S6 levels under basal (serum-starved) and FGF2-stimulated conditions as in (A). (**D**) NCI-H226 and MSTO-211H cells grown in full serum (10% FBS) conditions containing heparin sodium salt (10 μg/mL) were treated with DMSO (0.1%), GSK3052230 (15 μg/mL) or NVP-BGJ398 (500 nM) and lysates were harvested at the indicated time points for western blot analysis of phospho- and total ERK and S6 expression.

Inhibition of FGF/FGFR downstream signaling by GSK3052230 was tested in NCI-H226 and MSTO-211H mesothelioma cells stimulated with either FGF2 or other growth factors known to activate receptor tyrosine kinases (RTKs). GSK3052230 effectively inhibited MAPK signaling, evidenced by a reduction of phospho-ERK and phospho-S6 levels in FGF2-stimulated cells (Figure 2A). FGFR1 autophosphorylation and phospho-FRS2α (FGFR docking protein) expression were not detectable by western blot in these cells (not shown). Interestingly, while treatment with GSK3052230 caused a slight reduction in phosphorylation of EGFR and MET upon acute growth factor stimulation, no changes to downstream signaling occurred. Crosstalk between RTKs may occur, and previous work has demonstrated that inhibition of EGFR by gefitinib can also reduce phosphorylation of MET and HER3 [27]. To date, this type of crosstalk has not been observed with FGF/FGFR inhibitors. When GSK3052230 was compared with NVP-BGJ398, both molecules effectively inhibited phospho-ERK and phospho-S6 levels under serum-starved and FGF2-stimulated conditions (Figure 2B and 2C). The observation that basal phosphorylation levels were inhibited indicates that MAPK signaling in these cells is ligand-dependent. Similar results were observed in lung cancer cells that harbor FGFR1 amplification (Supplementary Figure 4). The effects of GSK3052230 and NVP-BGJ398 on FGF/FGFR downstream signaling were also tested under full serum conditions in a time course over a period of 24 hours. At all time points tested, phospho-ERK levels were reduced by both GSK3052230 and NVP-BGJ398 when compared to DMSO-treated cells (Figure 2D). In contrast, no changes to phospho-S6 levels were observed, suggesting that other signaling kinases can phosphorylate S6 protein under these conditions.

### GSK3052230 inhibits tumor growth of human mesothelioma xenografts in mice

**Figure 3:**
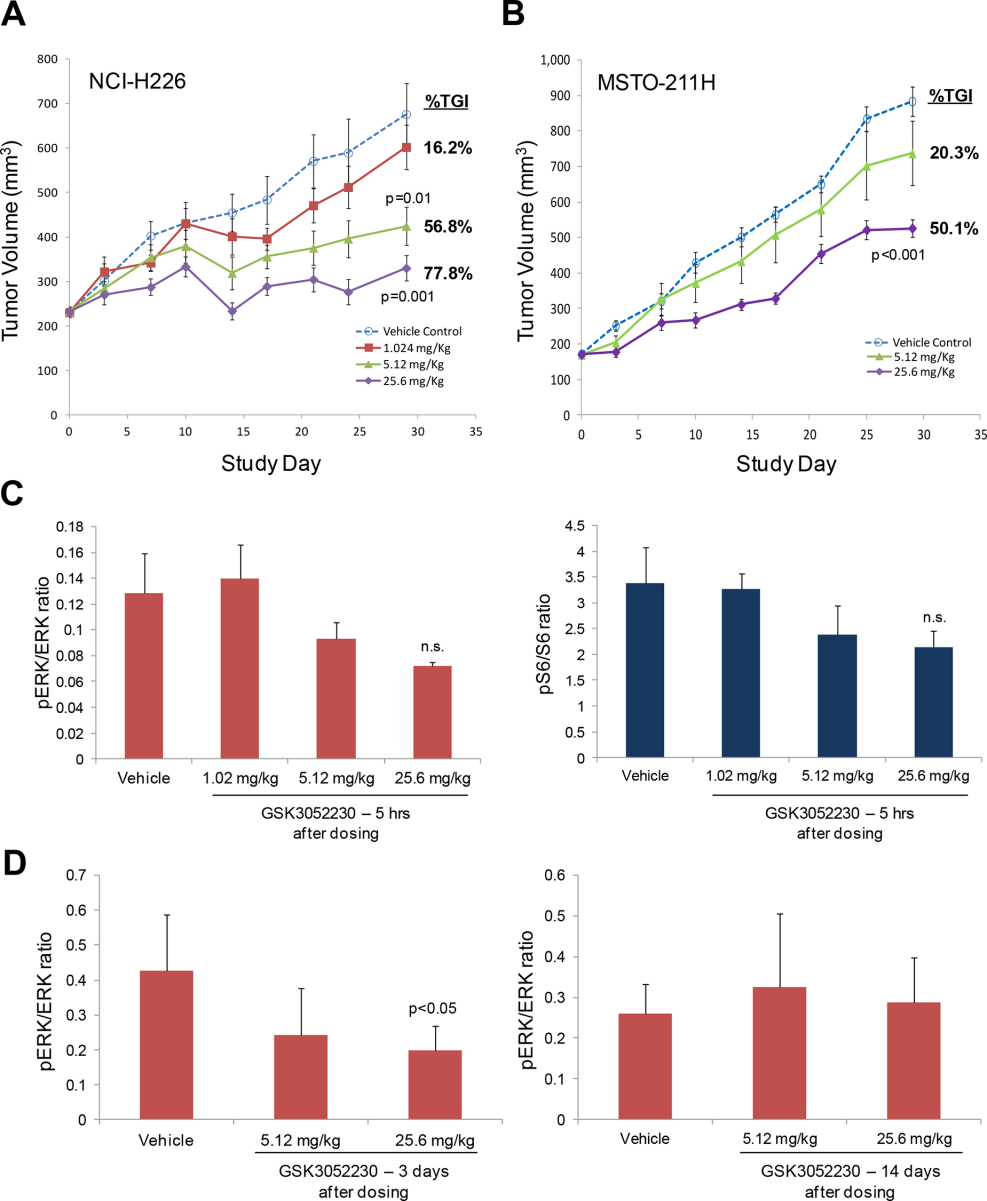
GSK3052230 inhibits tumor growth and MAPK signaling in mesothelioma xenograft models (**A**) Mice bearing subcutaneous NCI-H226 tumor xenografts (*n* = 8/group) were treated with vehicle or GSK3052230 at 1.024, 5.12 or 25.6 mg/kg three times per week for 4 weeks. The percentage of tumor growth inhibition (%TGI) observed at the end of the study (day 29) is shown here. Error bars correspond to the standard error of the mean (SEM); *p*-values were calculated by two sample (independent group) *t*-test assuming unequal variance. (**B**) Mice bearing subcutaneous MSTO-211H tumor xenografts (*n* = 10/group) were treated with vehicle or GSK3052230 at 5.12 or 25.6 mg/kg three times per week for 4 weeks. Measurements and data were collected as in (A). (**C**) Phospho-ERK/ERK and phospho-S6/S6 protein level ratios were determined by densitometry of western blot data from NCI-H226 tumors harvested five hours after the last dosing (day 29). Refer to Supplementary Figure 5A for the full western blot image. The observed reductions in phosphorylation of both proteins were not statistically significant (n.s.). Error bars correspond to standard deviation of triplicate samples. (**D**) Densitometry analysis of phospho-ERK/total ERK protein expression ratios in MSTO-211H tumors 3 days (left panel) and 14 days (right panel) after the last dosing. *p*-values were calculated by two sample (independent group) *t*-test assuming unequal variance.

To assess the effects of GSK3052230 on tumor growth, female SCID mice bearing subcutaneous NCI-H226 and MSTO-211H tumor xenografts were treated with vehicle or GSK3052230 at 1.024, 5.12, or 25.6 mg/kg three times per week for 4 weeks. GSK3052230 was well tolerated in both tumor models as assessed by body weight changes (Supplementary Figure 5). GSK3052230 administered at both 5.12 and 25.6 mg/kg caused significant tumor growth inhibition (TGI), 57% and 78%, respectively, compared to the vehicle control group in NCI-H226 tumor xenografts (Figure 3A). In MSTO-211H xenografts, GSK3052230 administered at 5.12 mg/kg did not significantly inhibit tumor growth (20% TGI), but the highest dose of GSK3052230 caused 50% TGI compared to the vehicle control group (Figure 3B). These results are consistent with the observed effects of this molecule *in vitro*, where NCI-H226 cells were more sensitive to GSK3052230 than MSTO-211H cells.

FGF/FGFR downstream signaling was explored by western blot analysis in NCI-H226 tumors (Supplementary Figure 6A). Phospho-ERK and phospho-S6 levels were modestly reduced in tumors treated with the two highest doses of GSK3052230 (Figure 3C). Similarly, several genes known to be activated by MAPK signaling were also tested by Taqman analysis to see if gene expression changes could be detected after GSK3052230 treatment. DUSP6, ETV1, and ETV4 mRNA levels were modestly reduced after GSK3052230 treatment, but none of the effects were statistically significant (Supplemental Figure 6B). The effects of GSK3052230 on phospho-ERK levels were also measured in MSTO-211H tumors that were harvested at 3 and 14 days after the last dose. Phospho-ERK levels were reduced by ~50% with GSK3052230 (25.6 mg/kg) treatment at day 3 but rebounded back to levels observed in vehicle-treated tumors after 14 days despite detectable levels of GSK3052230 in the tumors (Figure 3D; Supplementary Figure 6C). This data is consistent with the known short half-life of this molecule (~3.5 days) [28].

### Angiogenic effects of FGF pathway inhibition in GSK3052230-treated tumors

**Figure 4:**
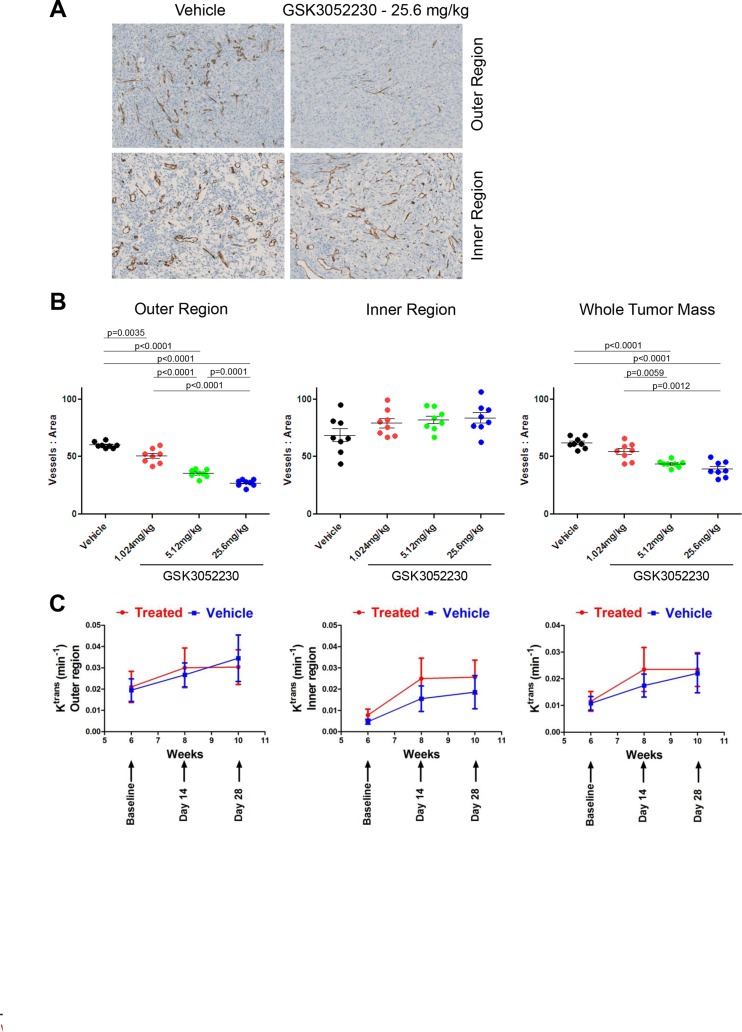
GSK3052230 reduces tumor vessel density in NCI-H226 xenografts (**A**) Representative photomicrographs of the outer and inner region of vehicle-treated or GSK3052230-treated NCI-H226 xenograft tumors stained for MECA-32 mouse endothelial cell protein by IHC. Qualitative review was indicative of two distinct morphological regions of the tumors (refer to Supplementary Figure 7A for representative image). (**B**) Quantification of MECA-32 IHC staining of NCI-H226 tumors from Figure 3A. Dose-dependent and statistically-significant reductions in tumor vessel density observed in the outer region and whole tumor mass data sets. (**C**) Effects of GSK3052230 on tumor vasculature permeability by DCE-MRI. Mice bearing subcutaneous NCI-H226 tumor xenografts (*n* = 10/group) were treated with vehicle (0.9% saline, blue line) or 25.6 mg/kg of GSK3052230 (treated, red line) by intraperitoneal (bolus) injection three times per week for 4 weeks. MRI was performed prior to treatment at baseline and post-treatment on days 14 and 28. Tumor segmentation and whole tumor mass analysis was performed. Results were presented as mean values and error bars correspond to the SEM.

To explore the effects of GSK3052230 on angiogenesis and more specifically, tumor vessel formation, NCI-H226 tumors were tested by immunohistochemistry (IHC) for expression of the mouse endothelial cell protein, MECA-32, upon GSK3052230 treatment (Figure 4A). Images of the stained tumors were separated into morphologically distinct outer and inner regions which were clearly observed upon MECA-32 detection (Supplementary Figure 7A). Quantification of MECA-32 staining was analyzed by measuring the number of vessels/area in the outer region, the inner region, and in the whole tumor mass. While no differences were found between groups for the inner region, blood vessel density was significantly lower in the GSK3052230-treated group compared to the vehicle group for both the outer region and the whole tumor mass (Figure 4B).

To get an understanding of how GSK3052230 treatment affects tumor blood flow and perfusion, dynamic contrast-enhanced magnetic resonance imaging (DCE-MRI) was performed. Mice were injected with a gadolinium-based contrast agent, and then the transfer constant (K^trans^) of contrast agent between the blood stream and the extracellular space was measured. In tissues where blood flow is adequate to deliver the contrast agent, K^trans^ represents the product of the endothelial permeability and endothelial surface area. K^trans^ measurements of NCI-H226 tumors showed no differences between GSK3052230-treated and vehicle-treated groups (Figure 4C, right panel). Upon closer examination of the tumors, K^trans^ maps showed a highly perfused area in the outer region of tumors compared to the center (Supplementary Figure 7B). A segmentation analysis was done to look at the different regions of the tumors, but despite this, no differences were found between treatment groups in both outer and inner segmented regions (Figure 4C). The ability of GSK3052230 to inhibit tumor vessel formation yet have no effect on blood flow and perfusion highlight the complexity and unique role of FGF biology in tumor angiogenesis.

## DISCUSSION

In this study, expression data taken from a broad panel of mesothelioma cells and lung cancer cell lines demonstrated that high levels of FGF2 and/or FGFR1 RNA expression correlated with the antiproliferative effects of two FGF pathway inhibitors that differ in their mechanism of action. There were two cell lines, however, that were exceptions to this observation. NCI-H1703 is a squamous non-small cell lung cancer (NSCLC) cell line that harbors both FGFR1 and PDGFRA amplifications [29]. Prior studies have demonstrated that this cell line is insensitive to FGF/FGFR inhibitors but will respond to kinase inhibitors that target the activity of multiple receptor tyrosine kinases [13, 25]. The other cell line, NCI-H2052, is a mesothelioma cell with high levels of FGF2 that previously demonstrated a lack of sensitivity to FGF pathway inhibition [26]. The reason for this cell line's lack of response to GSK3052230 or NVP-BGJ398 treatment is not known.

This study also demonstrated that GSK3052230 is effective in inhibiting tumor growth of FGF2/FGFR1-overexpressing mesothelioma xenografts. These effects on tumor growth are at least in part due to the ability of GSK3052230 to inhibit MAPK signaling as evidenced by decreased phospho-ERK and phospho-S6 levels *in vitro* and *in vivo.* In the tumor models, decreases in phospho-ERK protein levels and the mRNA levels of three genes downstream of ERK were observed as early as five hours after the last treatment. After three days of treatment at the highest dose of GSK3052230, a 50% decrease in phospho-ERK protein levels was observed. This demonstrates that partial inhibition of MAPK signaling is sufficient to delay tumor growth in mesothelioma, but complete inhibition of MAPK signaling and/or inhibition of additional survival pathways may be necessary to achieve complete inhibition of tumor growth or to achieve tumor regression. Combining GSK3052230 with other targeted therapies could address this concern. An additional caveat to consider is the possibility that other FGFs that are not inhibited or weakly inhibited by GSK3052230 could be secreted by the tumor or by cells in the tumor microenvironment and contribute to FGFR downstream signaling.

To further expand our knowledge of FGF biology in angiogenesis, we explored endothelial cell staining by IHC and tumor vascular permeability by DCE-MRI. In NCI-H226 tumor xenografts, dose-dependent reductions in tumor vessel density were observed by IHC, but the DCE-MRI data showed no effect of GSK3052230 on tumor blood flow and perfusion. Further, DCE-MRI measurements showed high perfusion in the outer region of the tumor which contrasted with the IHC results showing a significant reduction of blood vessels in the outer region of GSK3052230-treated tumors. The differences seen here between inner and outer regions of the tumor using two different approaches is not fully understood, yet clearly differs from the effects of VEGF/VEGFR antagonists on tumor angiogenesis and vascular function [30].

The data presented here supports the current clinical evaluation of GSK3052230 in previously untreated mesothelioma patients (NCT01868022). In this Phase 1b study, patient specimens will be retrospectively tested for increased FGF/FGFR expression to look for correlations with response to therapy [31]. Several small molecule FGFR inhibitors are also being tested in clinical trials [32–34]. In these studies, patients are pre-selected based upon the identification of FGFR genomic alterations taken from tumor biopsies. Our results and the results from other preclinical studies suggest that FGF2 and/or FGFR1 RNA overexpression could be used as an alternative biomarker approach to identify tumors that may respond to FGF pathway inhibitors [25, 26]. This would allow for broader testing of GSK3052230 across previously untested tumor types where FGF-FGFR autocrine signaling occurs and potentially provide additional benefit to patients with unmet clinical needs.

## MATERIALS AND METHODS

### Cell lines and reagents

Mesothelioma (MERO-14, MERO-41, MERO-82, NO36, ONE58, MSTO-211H, NCI-H226, NCI-H2052) and lung cancer (NCI-H1703, -H211, -H526, -H522, -H520, -H460, -H358, -H1395, -H2122, -H2023, -H1651, A549, SW1573, DMS-114, DMS-53) cell lines were obtained from ATCC (Manassas, VA), DSMZ (Braunschweig, Germany), or Sigma-Aldrich (St. Louis, MO). Cells were cultured in the appropriate culture medium supplemented with 10% fetal bovine serum (FBS) at 37°C in humidified incubators under 5% CO_2_. Heparin sodium salt and growth factors were purchased from Sigma-Aldrich. GSK3052230 is formulated in 0.94 mg/mL sodium phosphate monobasic, 1.9 mg/mL sodium phosphate dibasic, 8.8 mg/mL sodium chloride, 0.2 mg/mL polysorbate 80, pH 7.0 at a stock concentration of 12.8 mg/mL. NVP-BGJ398 was purchased from Selleck Chemicals (Houston, TX) and dissolved in DMSO at a stock concentration of 20 mM.

### Cell proliferation studies

Cells were cultured in 384-well plates (1 × 10^3^ cells/well) and treated with GSK3052230 (dose range: 606 nM − 1.2 pM) or NVP-BGJ398 (dose range: 28.2 μM − 53.8 pM) for 6 days in anchorage-independent methylcellulose (AIMC) media (0.6% final concentration of methylcellulose). Cell proliferation was measured using the CellTiter-Glo^®^ (CTG) Luminescent Cell Viability Assay (Promega, Madison, WI) according to the manufacturer's instructions. One cell plate was developed with CTG at the time of compound addition (T0 plate). Results were then expressed as a percentage of the T0 value (normalized to 100%) and plotted against the compound concentration after 6 days of treatment. The cellular response was determined by fitting the concentration response data using a 4-parameter curve fit equation and determining the concentration that inhibited cellular growth by 50% (gIC_50_).

### Tumor xenograft studies

Female CB-17 SCID mice (Taconic, Cambridge City, IN) were injected with 5.0 × 10^6^ cells to establish subcutaneous NCI-H226 or MSTO-211H tumor xenografts. Once tumors reached ~150–300 mm^3^, mice were randomized (*n* = 8/group for NCI-H226 study; *n* = 10/group for MSTO-211H study) and treated with vehicle (0.9% saline) or GSK3052230 at 1.024, 5.12 or 25.6 mg/kg by intraperitoneal (bolus) injection three times per week for 4 weeks. Tumor volume measurements and body weights were collected twice weekly. At the end of the study, all tumors were harvested and either flash frozen in liquid nitrogen or placed into 10% Buffered Formalin for RNA isolation and/or IHC staining. All animal studies were conducted in accordance with the GSK Policy on the Care, Welfare and Treatment of Laboratory Animals and were reviewed by the Institutional Animal Care and Use Committee at GSK.

### Western blot analysis

Freshly harvested cancer cells or xenograft tumors were lysed with 1X cell lysis buffer (Cell Signaling Technologies, Danvers, MA) containing protease and phosphatase inhibitors (Roche, Indianapolis, IN). Subsequently, 30–40 μg of protein was run on 4–12% Bis-Tris gels (Thermo Fisher, Waltham, MA), and protein was transferred onto nitrocellulose membranes (Thermo Fisher). Membranes were blocked for one hour using Licor Odyssey Blocking Buffer (Lincoln, NE) before immunoblotting using the following antibodies (all from Cell Signaling Technology, Danvers, MA, USA): pEGFR (#3777), total EGFR (#2239), pMet (#3077), total Met (#3127), pAKT (#4060), total AKT (#9272), pERK (#9101), total ERK (#4695), pS6 (#2211), and total S6 (#2317). Western blots were processed using Licor Odyssey Imaging System, and densitometry was performed using Licor Odyssey Imaging System software.

### RNA expression analysis

For the FGF family RNA expression analysis, cells were cultured in 6-well plates (1.75 × 10^5^ cells/well) and grown in AIMC media for 6 days prior to being harvested for RNA. Cells were collected and lysed in HTG Molecular lysis buffer (Tucson, AZ) according the manufacturer's instructions prior to being shipped to HTG Molecular for analysis. Relative RNA expression levels for the FGF family were measured using the HTG EdgeSeq Oncology Biomarker Panel Assay. For Taqman gene expression analysis of frozen tumors, RNA was isolated using Trizol reagent (Thermo Fisher) according to the manufacturer's recommendations. cDNA was generated using 1 μg of total RNA per reaction and the High-Capacity cDNA Reverse Transcription Kit (Thermo Fisher). RT-PCR was performed using a ViiA 7 real time PCR instrument (Thermo Fisher). The following primer/probe sets (all from Thermo Fisher) were used: DUSP6 (Hs04329643_s1), ETV1 (Hs0095195_m1), ETV4 (Hs00385910_m1), ETV5 (Hs00927557_m1), and β-actin (Hs99999903). Relative abundance was calculated using the relative standard curve method, which utilizes threshold cycle (Ct) values generated during PCR amplification. Target gene relative abundance was normalized to β-actin relative abundance.

### Immunohistochemistry and vessel density measurements

Tissue sections were deparaffinized, hydrated, and loaded on the Ventana Discovery Ultra system (Tucson, AZ). Antigen retrieval was performed using Tris-based (EDTA) buffer solution, CC1 (Ventana). Rat anti-mouse MECA-32 (BD Pharmingen, San Jose, CA), or IgG2a isotype control (Thermo Fisher) were incubated for 1 hour followed by detection using OmniMap anti-rat HRP and ChromaMap DAB detection kit (Ventana). Tissue sections were then counterstained with hematoxylin. Images were captured using a NanoZoomer slide scanner (Hamamatsu, Bridgewater, NJ), and whole section morphological regions were manually separated using Photoshop (Adobe Systems, San Jose, CA). The variable transitional border was excluded from the outer region analysis. Necrotic spaces with limited vessels and nuclei were also observed in a small number of tumors and were removed from inner and outer region analyses but not the whole tumor section analysis. Images were analyzed using Metamorph (Molecular Devices, Sunnyvale, CA). RGB thresholding and defined object area parameters were utilized to detect vessels, with vessel data being normalized to the associated tissue region areas. *p*-values were calculated by one-way ANOVA assuming unequal variance, and Bonferroni's multiple comparison test was used to adjust for the multiple comparisons.

### Dynamic contrast-enhanced magnetic resonance imaging

Tumor vasculature permeability was explored by DCE-MRI using a 9.4T/30 cm Bruker system (Billerica, MA) using a volume coil as described previously [35]. Female CB-17 SCID mice (Taconic) bearing subcutaneous NCI-H226 tumor xenografts (*n* = 10/group) were treated with vehicle (0.9%) or 25.6 mg/kg of GSK3052230 by intraperitoneal (bolus) injection three times per week for 4 weeks. MRI was performed prior to treatment at baseline and post-treatment on days 14 and 28. Image processing and data analysis were performed on a voxel-by-voxel basis using Jim 7 software (Xinapse Systems, Essex, UK). Tumor segmentation and whole tumor mass analysis was performed by measuring the transfer constant (K^trans^) of labeled contrast agent between the blood stream and the extracellular space surrounding the tumor.

## SUPPLEMENTARY MATERIALS FIGURES


