# Supplementary material for: Inhibition of FGF/FGFR autocrine signaling in mesothelioma with the FGF ligand trap, FP-1039/GSK3052230
Source: Oncotarget. 2016 May 20;7(26):39861–71. doi: 10.18632/oncotarget.9515 (PMC5129976; doi:10.18632/oncotarget.9515)
Supplement: Supplementary file 1 [file oncotarget-07-39861-s001.pdf]

Inhibition of FGF/FGFR autocrine signaling in mesothelioma with the FGF ligand trap, FP-1039/GSK3052230

Supplementary Materials

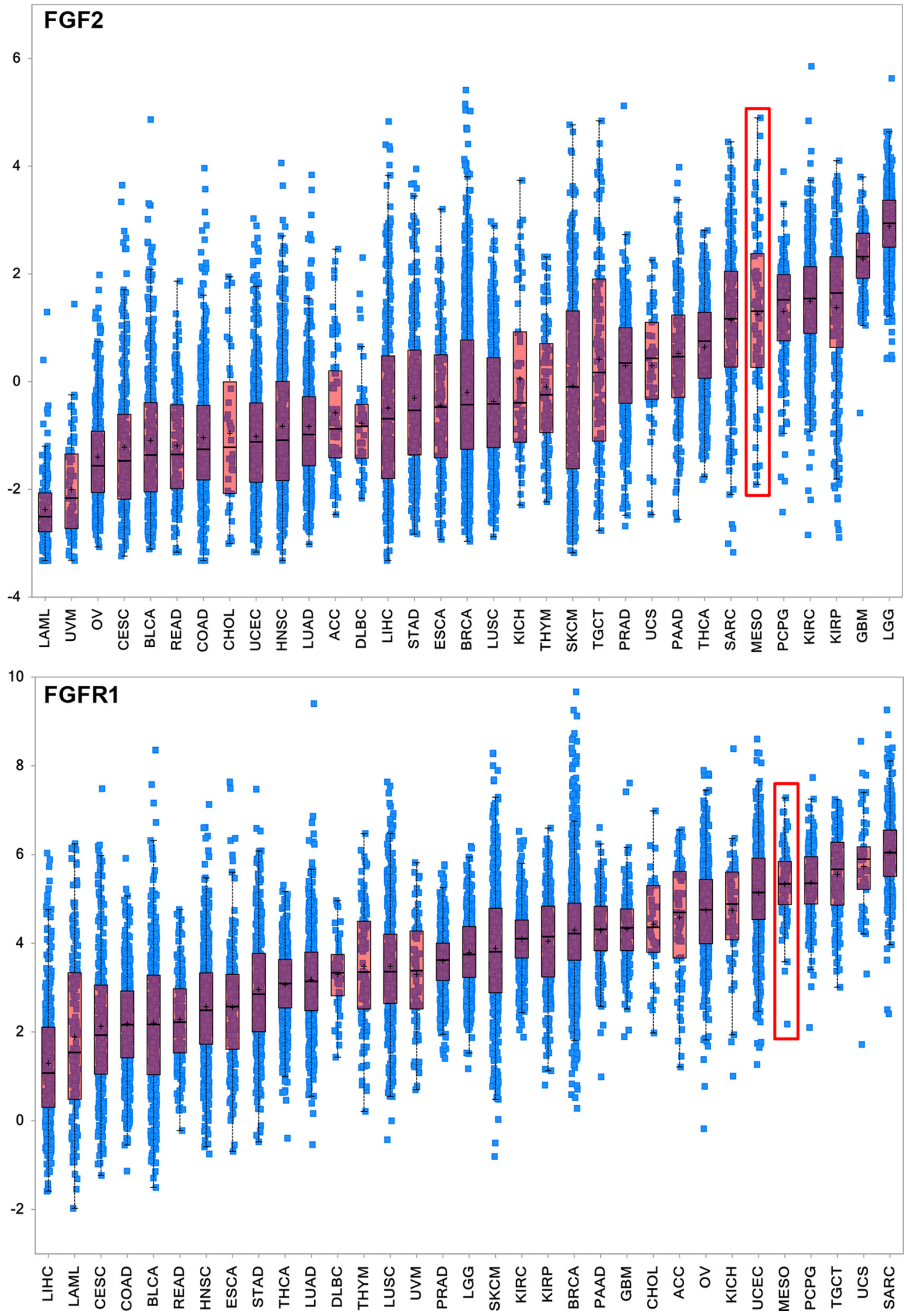

Supplementary Figure S1: Distribution of FGF2 (top) and FGFR1 (bottom) expression across 33 tumor types in The Cancer Genome Atlas (TCGA) collection. Log_2 transformed FPKM values (Y-axis) for tumor samples with RNA-seq data within each of the disease groups shown were plotted using the Omicsoft® OncoLand® 2015 Q1 release (Omicsoft, Cary, NC,

USA). ACC (adrenocortical carcinoma; *n* = 79); BLCA (bladder urothelial carcinoma; *n* = 430); BRCA (breast invasive carcinoma; *n* = 1227); CESC (cervical squamous cell carcinoma and endocervical adenocarcinoma; *n* = 309); CHOL (cholangiocarcinoma; *n* = 45); COAD (colon adenocarcinoma; *n* = 514); DLBC (diffuse large B-cell lymphoma; *n* = 48); ESCA (esophageal carcinoma; *n* = 198); GBM (glioblastoma multiforme; *n* = 174); HNSC (head and neck squamous cell carcinoma; *n* = 566); KICH (kidney chromophobe; *n* = 91); KIRC (kidney renal clear cell carcinoma; *n* = 610); KIRP (kidney renal papillary cell carcinoma; *n* = 323); LAML (acute myeloid leukemia; *n* = 179); LGG (lower grade glioma; *n* = 534); LIHC (hepatocellular carcinoma; *n* = 424); LUAD (lung adenocarcinoma; *n* = 589); LUSC (lung squamous cell carcinoma; *n* = 552); MESO (mesothelioma; *n* = 87); OV (ovarian serous cystadenocarcinoma; *n* = 430); PAAD (pancreatic adenocarcinoma; *n* = 183); PCPG (pheochromocytoma and paraganglioma; *n* = 187); PRAD (prostate adenocarcinoma; *n* = 554); READ (rectum adenocarcinoma; *n* = 177); SARC (sarcoma; *n* = 265); SKCM (skin cutaneous melanoma; *n* = 473); STAD (stomach adenocarcinoma; *n* = 457); TGCT (testicular germ cell tumors; *n* = 156); THCA (thyroid carcinoma; *n* = 572); THYM (Thymoma; *n* = 122); UCEC (uterine corpus endometrial carcinoma; *n* = 586); UCS (uterine carcinosarcoma; *n* = 57); UVM (uveal melanoma; *n* = 80).

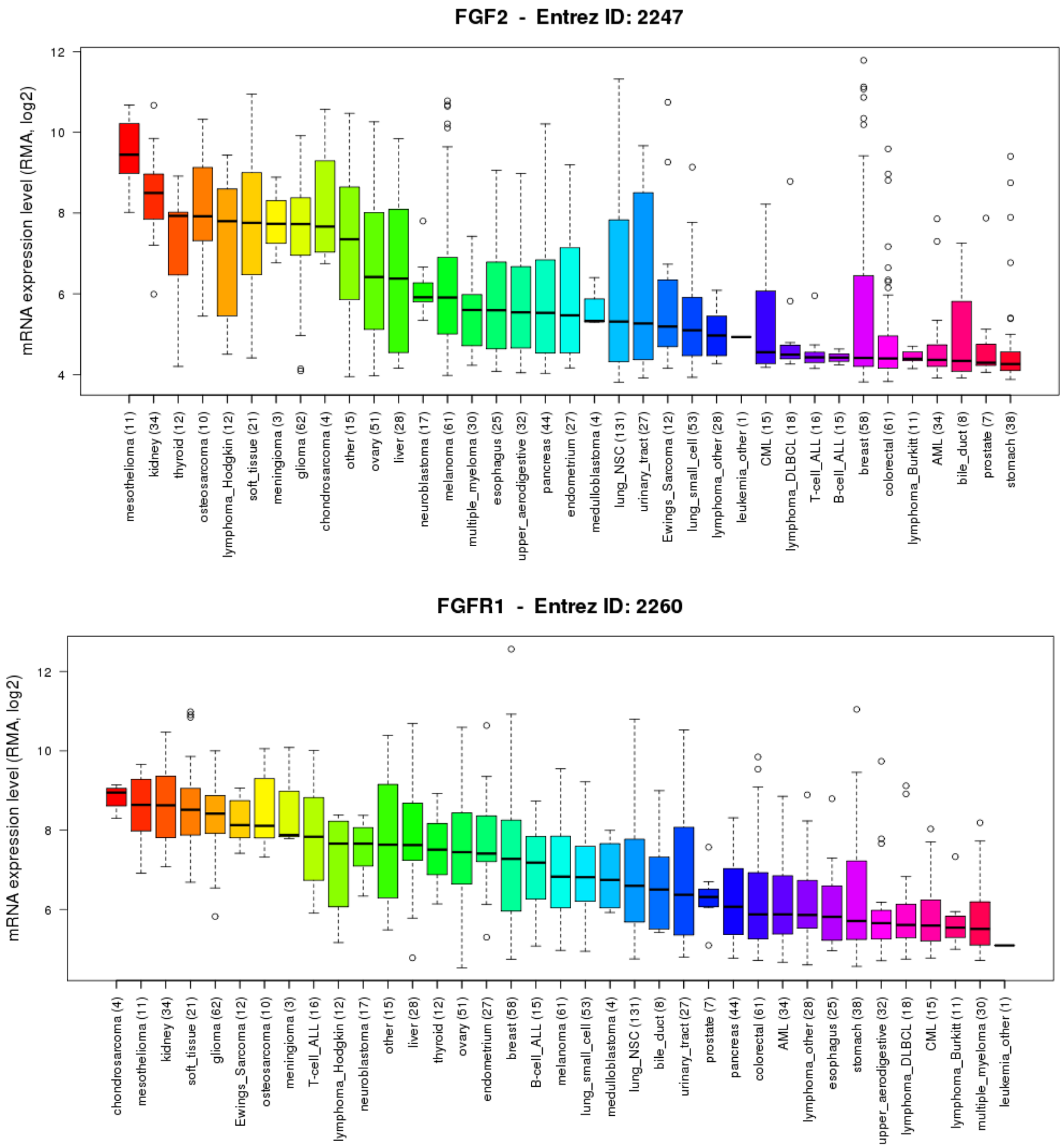

Supplementary Figure S2: FGF2 (top) and FGFR1 (bottom) expression across all cancer cell lines in the Cancer Cell Line Encyclopedia (CCLE). FGF2 mRNA expression levels are highest in mesothelioma cell lines, and FGFR1 levels are 2nd highest in mesothelioma cells across the CCLE panel (http://www.broadinstitute.org/ccle/home).

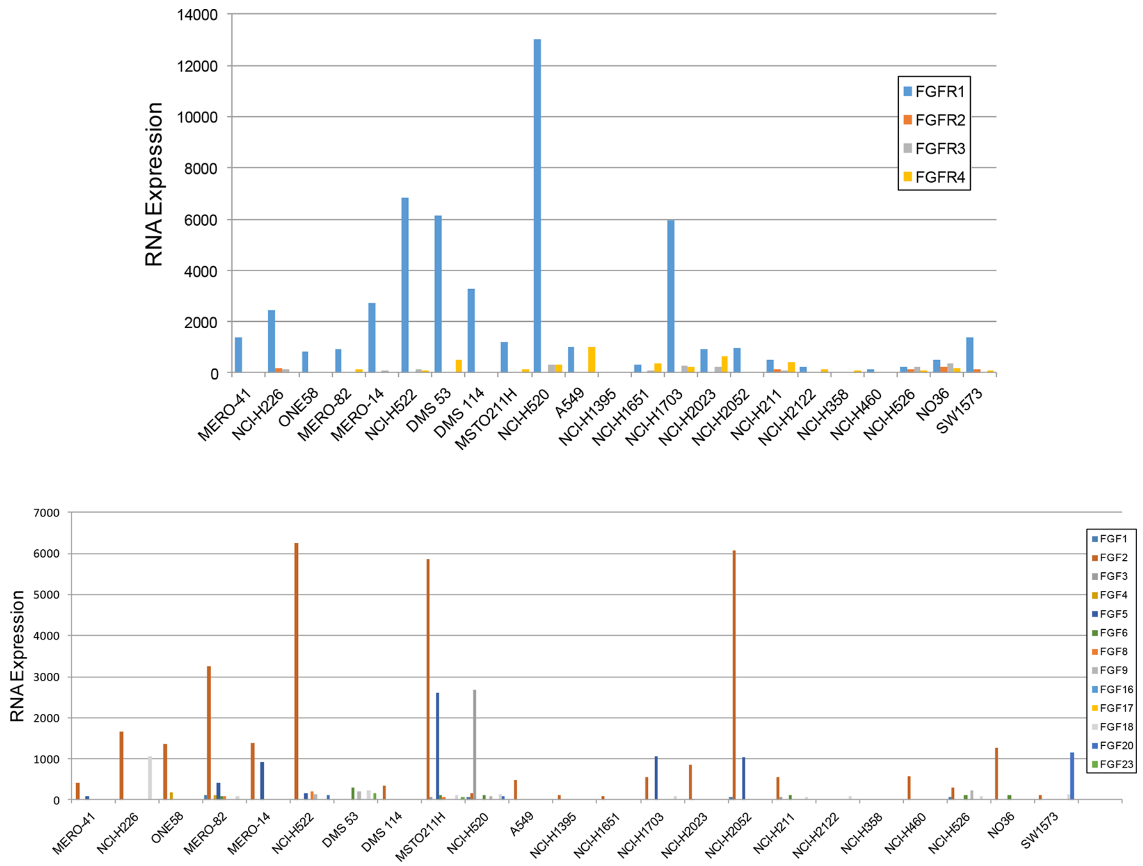

Supplementary Figure S3: FGFR1 and FGF2 are the most highly and broadly expressed FGF receptor and ligand, respectively, across the cell line panel. Baseline relative RNA expression for 23 mesothelioma and lung cancer cell lines was assayed using the HTG EdgeSeq Oncology Biomarker Panel Assay. For the FGF ligands (bottom panel), only those FGFs that have been shown to bind to GSK3052230 are presented here (refer to ref. 13).

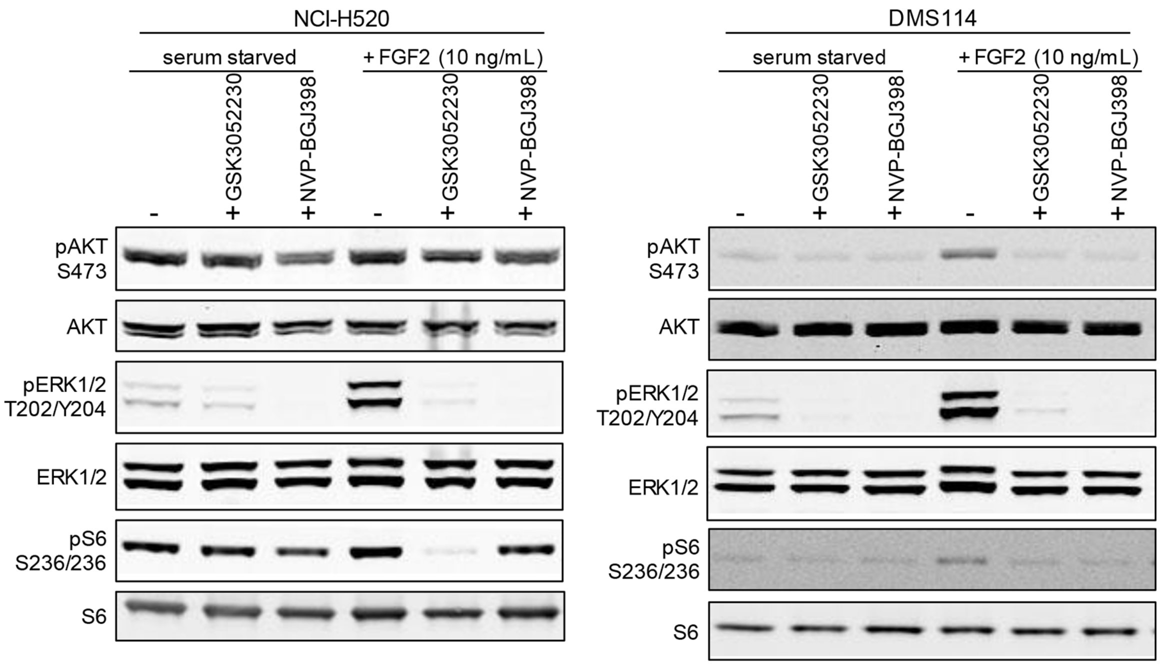

Supplementary Figure S4: PI3K/AKT and MAPK signaling after FGF2 stimulation in FGFR1-amplified lung cancer cell lines. Cells were serum starved for 24 hours and then pretreated with heparin sodium salt (10 µg/mL) with or without GSK3052230 (15 µg/mL) or NVP-BGJ398 (500 nM) for 2 hours prior to the addition of FGF2 for 15 minutes. Protein lysates were harvested and subjected to western blot analysis.

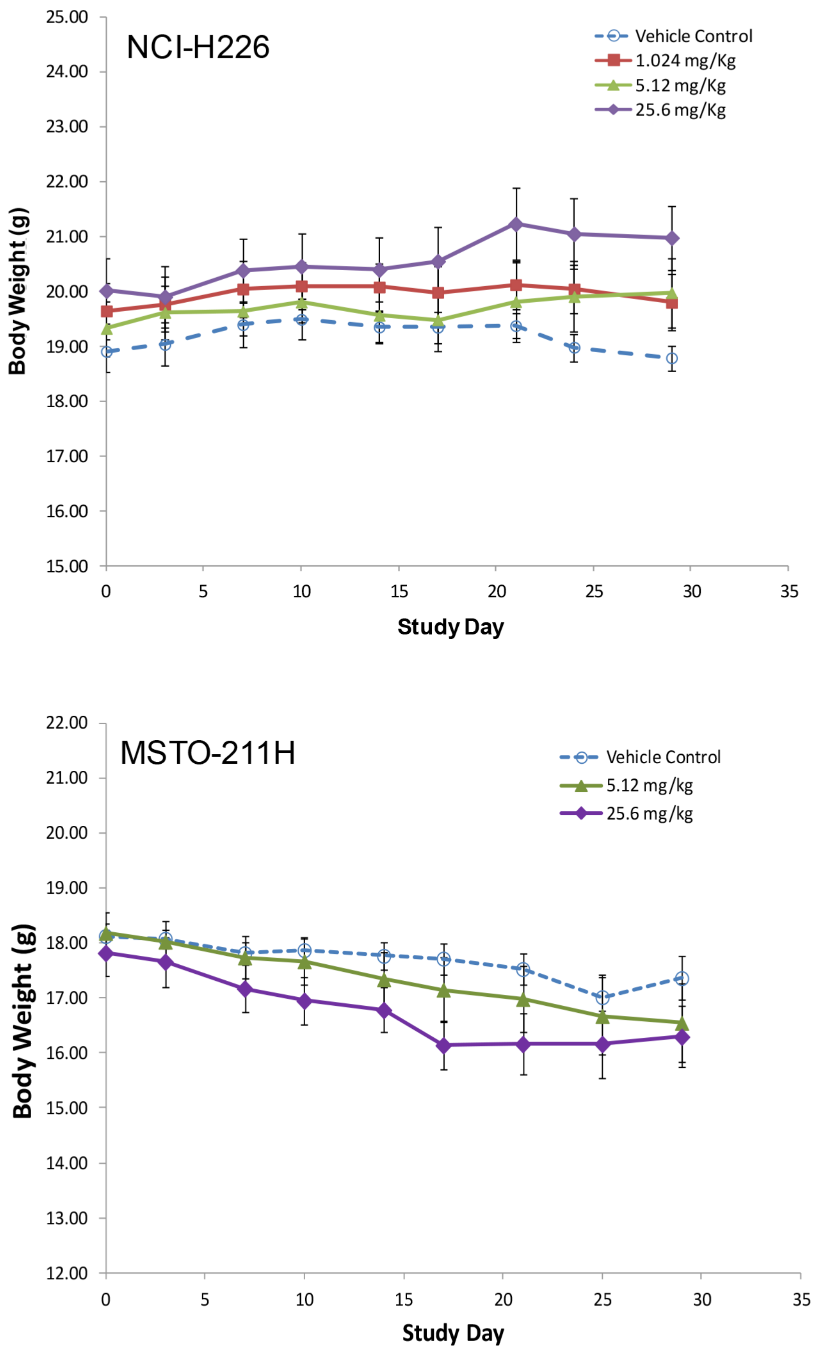

Supplementary Figure S5: Effects of GSK3052230 on body weight of mice bearing NCI-H226 (top) and MSTO-211H (bottom) xenografts. Female SCID mice bearing subcutaneous NCI-H226 (*n* = 8/group) and MSTO-211H (*n* = 10/group) tumor xenografts were treated with vehicle (0.9% saline) or GSK3052230 at 1.024, 5.12 or 25.6 mg/kg by intraperitoneal (bolus) injection three times per week for 4 weeks. Body weight data was collected twice a week. Error bars correspond to the standard error of the mean (SEM). All doses of GSK3052230 were well tolerated, as assessed by body weight changes. Refer to Figure 3 for tumor volume data.

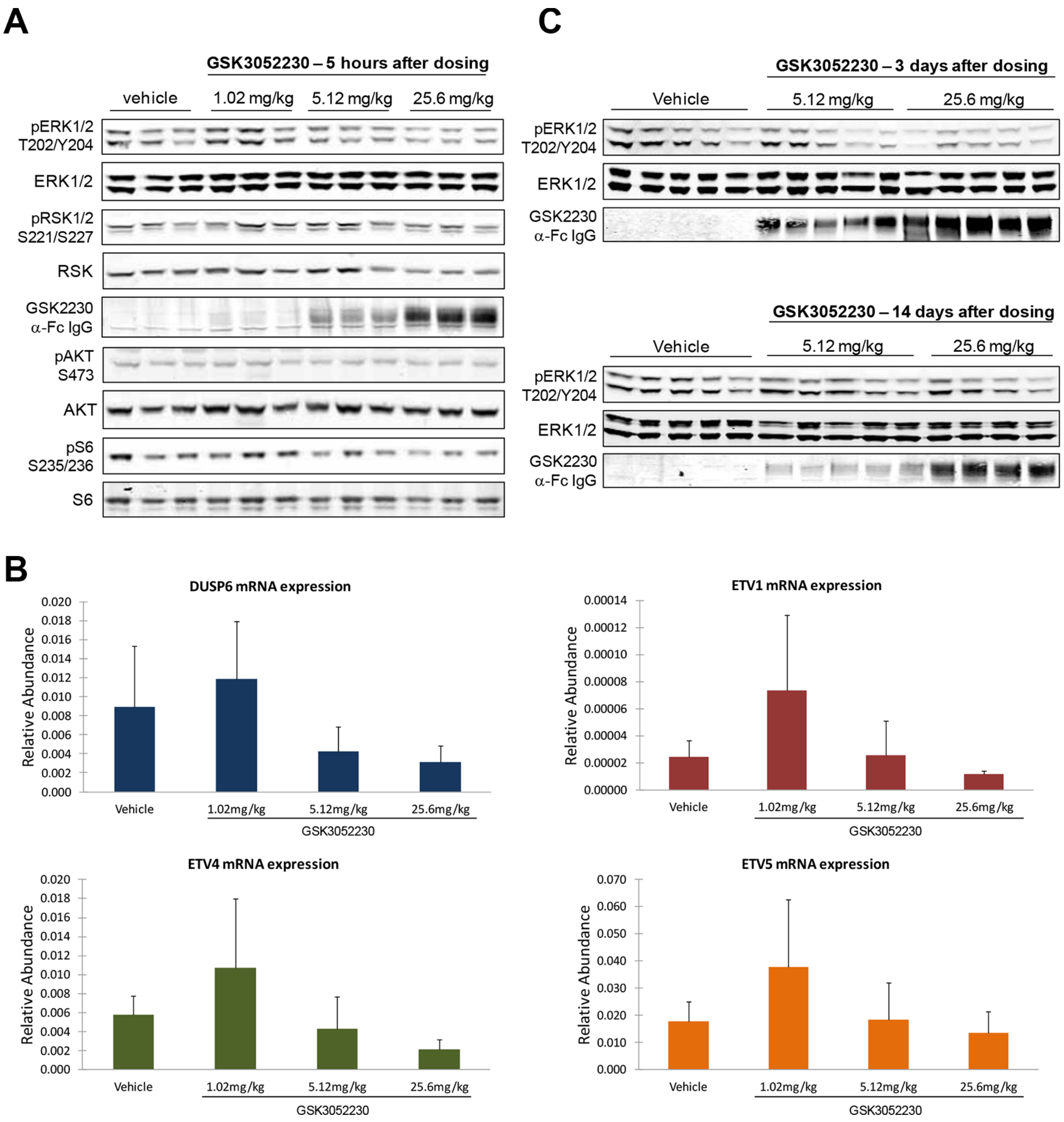

Supplementary Figure S6: Inhibition of MAPK signaling in NCI-H226 and MSTO-211H xenograft tumors observed by Western blot and Taqman analysis. (**A**) Western blot images used to perform densitometry of phospho-ERK/ERK and phospho-S6/S6 protein level ratios from NCI-H226 tumors in Figure 3C. (**B**) RNA was also collected from (A) to measure gene expression by Taqman analysis. Target gene relative abundance was normalized to β-actin relative abundance and plotted as bar graphs. Error bars correspond to standard deviation values of triplicate replicates. (**C**) Western blot images used to perform densitometry of phospho-ERK/ERK protein level ratios from MSTO-211H tumors in Figure 3D.

A

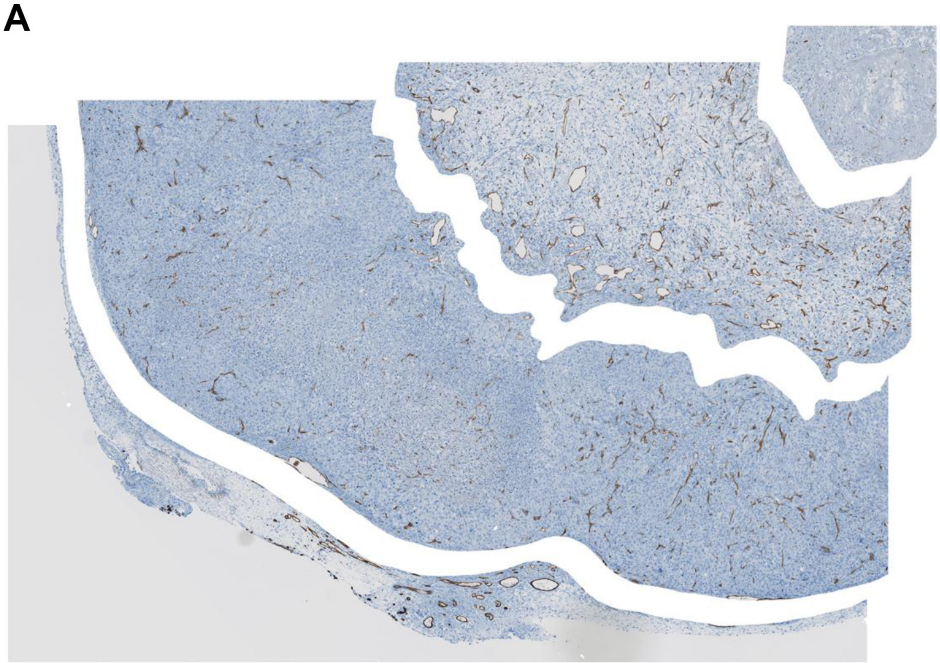

B

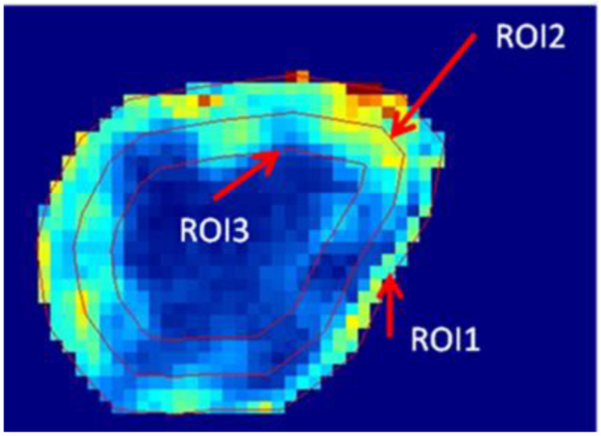

Supplementary Figure S7: Effects of GSK3052230 on angiogenesis. (**A**) Manual tumor region separation for MECA-32 IHC vessel density analysis. Qualitative review of MECA-32 IHC and H&E staining was indicative of two morphologically distinct inner and outer regions. In general, outer regions consisted of predominantly neoplastic cells and small areas of necrosis. Inner regions exhibited poor cellularity, oedema, fibroplasia, inflammatory cell infiltration including neutrophils & macrophages, and few neoplastic cells. At the transitional border between the two regions, considerable morphological variability was noted, including dense clusters of vessels and areas of elevated inflammatory infiltrate levels. (**B**) A segmentation analysis of the K^{trans} maps generated by DCE-MRI was performed to look at the different regions of NCI-H226 tumors. Mean K^{trans} values were measured for the whole tumor, the highly perfused area in the outer region (between ROI1-ROI2), and the inner region (between ROI2-ROI3). Representative images are shown here.
